# Relationships between Extra-School Tutoring Time, Somatic Symptoms, and Sleep Duration of Adolescent Students: A Panel Analysis Using Data from the Korean Children and Youth Panel Survey

**DOI:** 10.3390/ijerph17218037

**Published:** 2020-10-31

**Authors:** Jin-Won Noh, Jinseok Kim, Jooyoung Cheon, Yejin Lee, Young Dae Kwon

**Affiliations:** 1 Department of Health Administration, Dankook University, Cheonan 31116, Korea; health@dankook.ac.kr; 2 Department of Social Welfare, Seoul Women’s University, Seoul 01797, Korea; jskim@swu.ac.kr; 3 Department of Nursing Science, Sungshin Women’s University, Seoul 01133, Korea; jcheon@sungshin.ac.kr; 4 Department of Healthcare Management, Eulji University, Seongnam 13135, Korea; yiye1110@gmail.com; 5 Department of Humanities and Social Medicine, College of Medicine and Catholic Institute for Healthcare Management, The Catholic University of Korea, Seoul 06591, Korea

**Keywords:** sleep duration, adolescent students, tutoring time, somatic symptom

## Abstract

As private tutoring has expanded worldwide, it has been noted that private tutoring and associated emotional distress can affect sleep duration and the health of adolescent students. However, the relationships between extra-school tutoring time, somatic symptoms, defined as physical symptoms of emotional distress, and sleep duration in adolescents has rarely been determined. The aim of this study was to identify these relationships in adolescent students. Data from the Korean Children and Youth Panel Survey were analyzed to address the research questions. Weekday sleep duration, extra-school tutoring time, and somatic symptoms were measured using adolescents’ self-report questionnaires. A multilevel, structural equation model was utilized to test the relationships between these variables and was deemed appropriate considering the repeated measure of the panel data. After controlling for respondent sex, parent working status and education level, and family structure, adolescents’ extra-school tutoring time and level of somatic symptoms were associated with sleep duration during weekdays. Furthermore, the association between extra-school tutoring time and sleep duration was partially mediated by somatic symptoms. Korean adolescent students slept less than the recommended duration. Intervention programs that increase parental interest and attention in adolescent students’ lives, not only focused on academic achievement but also emotional distress is needed. Researchers and policymakers should understand recommended age-appropriate sleep duration and the educational culture and provide balanced strategies between the consideration of the effect of private education on academic achievement and the need to guarantee physical and mental health in adolescent students.

## 1. Introduction

Sleep patterns change throughout life [1], and childhood sleep patterns change dramatically from preadolescence to adolescence. As children transition from childhood to adolescence, marked changes occur in physiologic, psychologic, cognitive, and social domains, all of which have a substantial effect on sleep habits [2]. In adolescents, shorter sleep duration is associated with multiple indicators of adverse health status and a lower likelihood of better self-rated health (SRH) [3]. 

Previous findings have demonstrated that sleep deprivation may also be associated with poorer SRH [4], increased odds of being overweight/obesity [4,5,6], and a decrease in school performance [5,7,8]. Sleep deprivation also can lead to negative somatic symptoms, including impaired cognitive functioning [9], an increase in emotional instability [7,10], and depressive symptoms [4,5,11]. These somatic symptoms may be a trigger of the processes that produce and maintain somatic complaints in adolescents [12]. Chronically high perceived stress [13] and sleep deprivation [14] are associated with higher somatic complaints because school is being considered as the workplace of adolescents [13].

Despite these negative effects, modern-day adolescents get much less sleep than those in the 20th century [10]. Sleep duration and patterns during adolescence differ between countries, and Asian adolescents are more likely to fall asleep later than adolescents from Western countries, resulting in less total sleep time [15]. In particular, this situation is extreme in South Korea, where high school students reportedly average 4.9–6.5 h of sleep per day [2,10,16].

Most adolescent students do not get sufficient sleep due to competing demands of studying, social activities, the internet, and social networking [17,18,19]. Especially in countries where the participation rate for higher education is high and university education is relatively expensive, competition for entrance into a more prestigious university continuously expands the market for private tutoring. Private tutoring, called shadow education [20], has become a common phenomenon in many countries, including South Korea [21], Japan [22], Hong Kong [23], Turkey [24], and Greece [25], as participation in higher education becomes universal. The extra-school tutoring has become more common and perceived as initial elements of the adolescents and their families, even in developing countries, including Sri Lanka [26], Vietnam [27], and Egypt [28].

As private tutoring among adolescents has increased worldwide, education requirements become a burden, and quality of life is compromised [21,29]. Since high academic achievement is the most important goal for adolescent students in achieve-oriented cultures [30,31], other aspects of their development are largely ignored. This can seriously affect the overall quality of their lives [29]. Previous studies reported the current academic achievement-oriented society and its negative relationship to psychological well-being of adolescents [29,32,33]. Moreover, somatic symptom is related to short sleep duration in adolescents and has increased the risk of distress onset [34].

Therefore, there is increased interest between extra-school tutoring time and sleep duration of adolescent students. However, the relationships between extra-school tutoring time, somatic symptoms, and sleep duration in adolescent students have rarely been determined. Thus, the current study aimed to identify the relationships between extra-school tutoring time, somatic symptoms, and sleep duration of adolescent students through a panel analysis using nationally representative data.

## 2. Methods

### 2.1. Data and Participants

The Korean Children and Youth Panel Survey (KCYPS) was analyzed to address the research questions of this study. The KCYPS, based on a nationally representative sample of Korean children and youth, aimed to investigate various aspects of adolescents’ development. Three different age groups of children, first (age 6) and fourth grade (age 9) elementary and first grade junior high school students (age 12) in the first year of the study, were asked to answer a series of questions with a primary focus on growth and development and were followed up for the next six years. The KCYPS started its annual data collection in 2010 and continued until 2016. The KCYPS employed a multi-stage stratified cluster sampling method with school as the primary sampling unit. The data from the panel of the first grade junior high school students (*n* = 2351) across 78 schools who were followed up until their last year of high school were used in this analysis. At the sixth wave of the data collection, the original sample retention rate of the first-year junior high school students’ panel was 87.5% (*n* = 2056 at Wave 6). This study was approved by the Institutional Review Board of Seoul Women’s University (IRB-2018-46) with a waiver for informed consent because the data were obtained from a public data depository freely accessible online [35].

### 2.2. Variables and Measurements

Adolescent sleep duration was calculated based on the bedtime and wake-up times of the respondents. These times were measured using the following question: “What times do you usually fall asleep and get up?” This question was asked separately for weekdays and weekend days, although our study analyzed responses of weekdays only.

Adolescent somatic symptoms, defined as physical symptoms of emotional distress [36], was measured using eight questions about their experiences of sleeplessness, headache, nausea, stomachache, loss of appetite, fatigue, difficulty breathing, and fever. The respondents were asked to rate each variable using a four-point Likert scale, and the average score was used in the analysis. These questions were asked in Waves 2, 3, 4, 6, and 7 in the KCYPS, for which Cronbach’s alpha ranged from 0.818 (Wave 4) to 0.875 (Wave 3).

A series of questions was asked of respondents to measure daily time use pattern. Specifically, this analysis utilized a measure of time spent on extra-school tutoring classes plus assignments for extra-school tutoring classes. These questions were also asked for weekdays and weekends separately, though only weekday responses were used in our analysis.

Adolescent demographic and socio-economic characteristics were included in this analysis. Socio-economic status was measured by parental working status and parental education level. Working status was asked of both parents and used as separate variables. Parent education level was defined based on college graduation, and those of mothers and fathers were used as separate variables. Family type was operationalized as a single parent family or other type of family, with a two-parent family as a reference.

### 2.3. Statistical Analysis

A series of descriptive analyses was conducted to provide overall characteristics of the sample. Considering the repeated measure of the panel data, a multilevel structural equation model (SEM) was utilized to test the model relationships between extra-school tutoring time, psychological distress, and sleep duration of Korean adolescents. The multilevel SEM model can be written as follows:

$${\left[sleep\right]}_{ij}=\beta_{0j}^{1}+\beta_{1j}^{1}{\left[wave\right]}_{ij}+\beta_{2j}^{1}{\left[somatic\;symptoms\right]}_{ij}+\beta_{3j}^{1}{\left[tutoring\;hours\right]}_{ij}+\overset{10}{\underset{k=4}{\sum }}\beta_{kj}^{1}{\left[covariates\right]}_{ij}^{k}+\varepsilon_{ij}^{1}$$

$${\left[somatic\;sysmptom\right]}_{ij}=\beta_{0j}^{2}+\beta_{1j}^{2}{\left[tutoring\;hours\right]}_{ij}+\varepsilon_{ij}^{2},$$

where $\beta_{0j}^{m}=\gamma_{00}^{m}+u_{0j}^{m};\;u_{0j}^{m}\sim N\left(0,\tau_{00}^{m}\right);\;\beta_{lj}^{m}=\gamma_{00}^{m}\;\mathrm{if}\;l\neq 0,$ and $\varepsilon_{0j}^{m}\sim N\left(0,{\left(\sigma^{m}\right)}^{2}\right)$, here m=1, 2.

Missing values in the data were treated using an equation wise deleter (StataCorp, College Station, TX, USA). A multilevel approach was necessary to account for the repeated measure of the panel data in the analysis [37]. Stata Statistical Software Release 15 (StataCorp, College Station, TX, USA) was used to manage the data and Mplus 8.0 (Muthén & Muthén, Los Angeles, CA, USA) was used to analyze the multilevel model.

## 3. Results

A summary of sample characteristics is presented in Table 1. About the same number of females as males participated throughout the study period. The proportion of working mothers ranged from 62.5 to 70.9%, while that of fathers was greater than 90%. In terms of education level, the proportion of mothers with a college degree or higher ranged from 26.0 to 32.8%. The proportion of fathers with a college degree or higher ranged from 40.7 to 45.1%. Most of the participants were from a two-parent household, while slightly less than 10% were from a single-parent household (minimum: 7.9%, maximum: 9.5%). Similar numbers of adolescents were from different family types such as living with grandparents (minimum: 7.1%, maximum: 12.3%).

On average, Korean adolescent students slept from about 367 (mean (M) (standard deviation (SD)) = 367.0 (67.0) in Wave 5 or the second year in high school) to 474 min (M (SD) = 474.1 (58.8) in Wave 1 or the first year in junior high school) during the study period. This change of sleep duration corresponds to 13.9 min decrease per year on the average during the study period. They spent from about 1.1 (M (SD) = 66.9 (115.6) minutes in Wave 6 or the third year in high school) to 3.8 h (M (SD) = 165.4 (126.0) minutes in Wave 1) on extra-school tutoring classes and assignments during the same period (Table 1).

**Figure 1:**
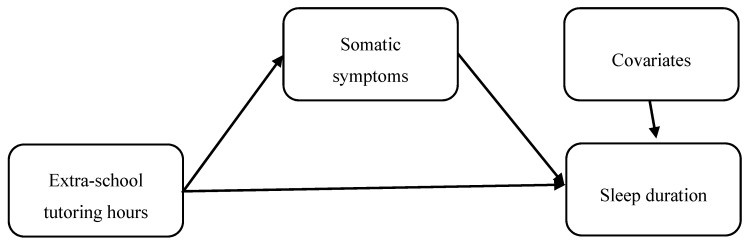
Illustration of the analysis model.

Table 2 summarizes the analysis results of the multilevel SEM (Figure 1). Overall, the results showed that the time Korean adolescent students spend on tutoring activities outside school impacted sleep duration not only directly, but also indirectly measured by level of somatic symptoms related with emotional distress/problem. The results showed that, after controlling for respondent sex, parental working status, parental education level, and family type, the hours spent on tutoring outside school (B (standard error (SE)) = −2.06 (0.41), *p* < 0.001) and adolescent level of somatic symptoms (B (SE) = −6.79 (1.48), *p* < 0.001) were negatively associated with sleep duration. Also, those who spent more hours on tutoring outside school reported higher levels of somatic symptoms (B (SE) = 0.01 (< 0.01), *p* = 0.010). Further, the test of the indirect effect of time spent on extra-school tutoring activities on sleep duration via somatic symptoms related with emotional distress/problem showed a significantly negative value (B (SE) = −0.06 (0.03), *p* = 0.024) (Table 2).

## 4. Discussion

In this nationally representative sample, adolescent students who spent more time on tutoring activities outside school showed higher levels of somatic symptoms, which ultimately resulted in shorter sleep duration. Korean adolescent students reported that they slept for an average of 6.1–7.9 h on weekdays, 7.4–7.9 h for middle school students and an average of 6.1–6.3 h for high school students. This supports the idea that high school students suffer greater sleep deprivation [4,10]. The National Sleep Foundation recommends a sleep duration of 9–11 h for younger school age children (six to 13 years old) and 8–10 h for teenagers (14–17 years old) [38], while other studies recommended sleep duration of 9–11 h among adolescents [39,40]. According to the findings of a systematic review to examine age-related sleep duration, estimates for nighttime sleep duration on weekdays were 7.8 h in those 12–14 years old and 7.88 h in the 15–18-year-old age group [41]. Korean adolescent students slept less than those in other countries.

In this study, time spent on tutoring activities outside schools was a significant factor associated with sleep duration. One of the distinctive characteristics of Korean education is “educational zeal,” representing an extraordinary degree of parental interest in children’s educational achievement [42,43]. Because students with a high score usually have a greater chance of getting into top universities, private tutoring activities may play a substantial role for achieving academic success in Korea. Korean Statistics (2019) reported that 72.8% of school-age children and teenagers participated in extra-school activities [44]. It is common for Korean students to study after school at private institutions or with private tutors, as was demonstrated in our study findings, in which adolescent students reported spending an average of 1.1–3.8 weekday hours on extra-school tutoring classes and assignments.

Rhie et al. (2011) reported that, despite internet-attributed sleep deprivation among adolescents, excessive tutoring time was a major cause of sleep deprivation [10]. Early school start time and longer study time resulting in later bedtime were also considered major barriers to improving sleep duration and quality [4,45]. Unfortunately, the campaign for delayed school start time in South Korea only transiently increased sleep duration. It was not sustained because adolescent students still had too many extracurricular activities, homework, and private tutoring activities after school [46]. Therefore, further interventions should focus on strategies for the improvement of public education to reduce private tutoring activities. For example, the development of a personalized online/offline education programs, especially focused on English and Mathematics, which accounts for most of private tutoring expenses, may help students use their school time as efficiently as possible [47].

Adolescence is a critical period hallmarked by dramatic maturational changes that are associated with sleep duration and quality. Consistent with previous findings that somatic complaints were associated with sleep alteration in adolescence, especially in female students [14,48], somatic symptoms were positively associated with tutoring hours and negatively associated with sleep duration in this study. The significant finding of this study demonstrated the mediating effect of somatic symptoms in the relationship between extracurricular education and sleep duration. Somatic complaints are common in adolescence and may be related to emotional distress due to the pressure to improve academic achievement, especially in the distinctive Korean educational culture [14,42,43].

Even though time and efforts for private tutoring may have a negative effect on somatic symptoms and sleep at night, simply relieving somatic symptoms is not a fundamental solution to improve sleep duration because both parents and adolescent students are blind to private education for entering top-notch universities in a highly competitive Korean society [14,42,43,48]. A few studies have shown that private education mainly had a short-term effect on improving academic achievement, and the effect of private education on academic achievement declined with higher grade levels, and the effect was significant only in the group of low-achieving students in Korea [49,50]. Another study described that extra study time disrupt students’ sleep patterns, which may lead to academic problems [8]. Therefore, further longitudinal research is needed to analyze why Korean parents and adolescent students rely on private education, how effective private education is, and how to relieve emotional stress from private education. Also, there is an urgent need to prepare social conditions to alleviate blindness to private education and improve adolescent students’ emotional distress. Educational programs and campaigns that the ‘success’ should not be limited to educational achievement but should be extended to a wider range of success could be provided to both parents and adolescent students.

Interestingly, parental education level was associated with shorter sleep duration [51]. This may be due to Korean parents’ extraordinary interest in their children’s academic achievement and parental involvement including private tutoring time, which may be related to sleep alterations in adolescent students [42,43,52]. More highly educated parents had more discussion with their children and were involved in more private tutoring-related activities than those with lower educational levels [42,53]. Korean parents believe that education is extremely important, and that studying hard contributes to success [53]. In the context of Korean culture, intervention programs that increase parental interest and attention in children’s lives, not only focused on academic achievement but also emotional health and well-being should be investigated because parents play a critical role in their children’s health and quality of life and they should not project their faith and wishes toward ‘success’ to their children.

This study has some limitations. First, the analysis included adolescents who reported extra-school tutoring classes and/or assignments for such classes, which may result in exclusion of some applicable adolescents who did not answer the question (selection bias). This lack of response may be due to educational beliefs or parents’ socio-economic status, which were not represented in this analysis. Second, other factors, such as time spent in internet use and mobile phone and quality of sleep were not considered in this study. Thus, the relationship of time spent in internet use and mobile phone with sleep duration was not determined. Third, self-reported answers about sleep time and time spent in tutoring activities are related to recall bias and reporting bias. More objective ways of measuring time are needed, and the instrument used in this study would be validated in future studies. Fourth, we could not cover the somatic symptoms in Waves 1 and 5. It may affect the SEM outcomes. In addition, this study utilized only weekday responses of time spent on extra-school tutoring classes. Thus, future study considering both weekdays and weekend is needed to identify the important factors leading to poor health in adolescent students. Lastly, reverse causality might occur between sleep duration and somatic symptoms.

## 5. Conclusions

In this nationally representative sample, extra-school tutoring time was directly related to shorter sleep duration, and somatic symptoms mediated the effect of extra-school tutoring time on sleep duration. Private tutoring is obviously a contributor to academic achievement in Korea, but sufficient sleep is also important for academic achievement and health outcomes. Even though students should not sacrifice sleep to study, it is not easy to sleep sufficiently in highly competitive educational environments. Parents, educators, researchers, and policymakers should understand recommended age-appropriate sleep duration and the educational culture, and provide balanced strategies between the consideration of the effect of private education on academic achievement and the need to guarantee physical and mental health in adolescent students.

## Figures and Tables

**Table 1:** Socio-demographic and sleep related characteristics of the sample of adolescent students (*n* = 2351).

Variable	Wave 1 (*n* = 2351)		Wave 2 (*n* = 2280)		Wave 3 (*n* = 2259)		Wave 4 (*n* = 2108)		Wave 5 (*n* = 2091)		Wave 6 (*n* = 2056)	
	*n*	%	*n*	%	*n*	%	*n*	%	*n*	%	*n*	%
Sex: female	1175	50.0	1128	49.5	1119	49.5	1033	49.0	1024	49.0	1015	49.4
Working mother	1376	62.5	1401	67.3	1422	68.3	1350	68.3	1346	70.9	1269	65.1
Working father	2101	96.5	2018	98.4	1999	97.4	1909	97.4	1820	97.5	1761	90.4
Mother college graduate	571	26.0	676	32.6	676	32.4	642	32.5	619	32.6	602	32.8
Father college graduate	883	40.7	944	46.2	931	45.3	884	45.1	840	45.0	801	44.6
Family type: single parent	224	9.5	176	7.9	207	9.3	195	9.2	191	9.4	180	9.2
Family type: other	288	12.3	232	10.5	188	8.5	166	7.9	152	7.5	139	7.1
	Mean	SD	Mean	SD	Mean	SD	Mean	SD	Mean	SD	Mean	SD
Sleep duration (minute)	474.1	58.8	460.3	57.9	441.4	60.4	378.8	65.8	367.0	67.0	378.8	72.4
Somatic symptoms			2.0	0.6	2.1	0.6	2.0	0.5			1.9	0.6
Extra-school tutoring (minute)	165.4	126.0	149.9	119.4	126.6	112.6	76.0	101.5	75.4	103.8	66.9	115.6

SD—standard deviation.

**Table 2:** Multilevel structural equation model results: relationships between extra-school tutoring time, somatic symptoms, and sleep duration of adolescent students.

Dependent Variables	Independent Variables	B	SE (B)	95% CI	β ^a^
Direct effects					
Sleep duration					
	Somatic symptoms	−6.79 ***	1.48	(−9.69, −3.89)	−0.045
	Extra-school tutoring hours	−2.06 ***	0.356	(−2.76, −1.37)	−0.061
	Wave	−24.53 ***	0.411	(−25.33, −23.72)	−0.609
	Sex-female	−16.36 ***	1.803	(−19.89, −12.82)	−0.238
	Working mother	−2.08	1.608	(−1.07, 5.24)	0.030
	Working father	−1.17	5.129	(−11.22, 8.88)	−0.017
	Family type-single parent	35.22 **	11.472	(12.74, 57.71)	0.513
	Family type-other	2.54	3.114	(−3.56, 8.65)	0.037
	Father college graduate or higher	−5.20 *	2.077	(−9.27, −1.13)	−0.076
	Mother college graduate or higher	−7.84 ***	2.151	(−12.06, −3.63)	−0.114
Somatic symptoms					
	Extra-school tutoring hours	0.01 *	0.00	(0, 0.01)	0.020
Indirect effects					
Sleep duration					
	← [Somatic symptoms] ←[Extra-school tutoring hours]	−0.06 *	0.03	(−0.11, −0.01)	
R-squared	Sleep duration	0.378 ***			
	Somatic symptoms	0.002			

SE—standard error; CI—confidence interval; * *p* < 0.05; ** *p* < 0.01; *** *p* < 0.001; ^a^ standardized coefficient.
